# The Effects of Supplementary Food on the Breeding Performance of Eurasian Reed Warblers *Acrocephalus scirpaceus*; Implications for Climate Change Impacts

**DOI:** 10.1371/journal.pone.0159933

**Published:** 2016-07-28

**Authors:** James O. Vafidis, Ian P. Vaughan, T. Hefin Jones, Richard J. Facey, Rob Parry, Robert J. Thomas

**Affiliations:** 1 Cardiff School of Biosciences, The Sir Martin Evans Building, Cardiff University, Cardiff, CF10 3AX, United Kingdom; 2 Wildlife Trust of South and West Wales, Parc Slip, Tondu, Bridgend, CF32 0EW, United Kingdom; Cornell University, UNITED STATES

## Abstract

Understanding the mechanisms by which climate variation can drive population changes requires information linking climate, local conditions, trophic resources, behaviour and demography. Climate change alters the seasonal pattern of emergence and abundance of invertebrate populations, which may have important consequences for the breeding performance and population change of insectivorous birds. In this study, we examine the role of food availability in driving behavioural changes in an insectivorous migratory songbird; the Eurasian reed warbler *Acrocephalus scirpaceus*. We use a feeding experiment to examine the effect of increased food supply on different components of breeding behaviour and first-brood productivity, over three breeding seasons (2012–2014). Reed warblers respond to food-supplementation by advancing their laying date by up to 5.6 days. Incubation periods are shorter in supplemented groups during the warmest mean spring temperatures. Nestling growth rates are increased in nests provisioned by supplemented parents. In addition, nest predation is reduced, possibly because supplemented adults spend more time at the nest and faster nestling growth reduces the period of vulnerability of eggs and nestlings to predators (and brood parasites). The net effect of these changes is to advance the fledging completion date and to increase the overall productivity of the first brood for supplemented birds. European populations of reed warblers are currently increasing; our results suggest that advancing spring phenology, leading to increased food availability early in the breeding season, could account for this change by facilitating higher productivity. Furthermore, the earlier brood completion potentially allows multiple breeding attempts. This study identifies the likely trophic and behavioural mechanisms by which climate-driven changes in invertebrate phenology and abundance may lead to changes in breeding phenology, nest survival and net reproductive performance of insectivorous birds.

## Introduction

A central task for ecologists is to understand the drivers of population change and community structure. Food availability is a central consideration and understanding food limitation in natural populations helps to inform the impacts of environmental change on animal populations. In this study, we investigate the mechanisms linking changes in food availability with changes in the breeding behaviour and productivity of a bird population. Migratory insectivorous songbirds are well-studied and respond to short-term changes in the environment [1]. For example, in temperate regions, climate change is advancing the emergence dates of invertebrates in spring, so that food for many breeding insectivorous songbirds peaks earlier in the breeding season [2–5]. As income breeders, whose breeding productivity depends primarily on current resources [6], songbirds require large quantities of food to support the energetically costly activities of egg production, incubation and nestling provisioning. The breeding season is therefore a period when birds are strongly affected by changes in food abundance, and in the timing of food availability.

Many songbird species have shown advances in mean laying date in response to warming spring temperatures, which are likely to be linked to phenological advances in their prey [7–11]. Failure to adapt to this shift due to phenological mismatch has resulted in population declines in some long-distance migrant species reliant on short-lived peaks in invertebrate abundance early in the breeding season[12–14]. The influence of phenological mismatch, however, may have less impact on population sizes than widely believed [15]. Instead, the abundance of prey, rather than timing of prey peaks has been suggested as a more important consideration for breeding [16,17]. In wetlands, where the peaks of invertebrate abundance are more prolonged, the effects on breeding birds of climate-driven advancement of prey phenology may be less marked or even beneficial [11].

In many songbird species, increases in food availability are associated with larger clutch sizes [18,19] and with earlier laying dates [20–25]. In multi-brooding species, earlier initiation and completion of the first brood can result in a greater likelihood of successful completion of the second brood, and higher overall productivity [26, 27]. However, while larger clutches potentially increase first brood productivity by producing more offspring, the larger clutches take longer to lay and incubate [28,29]. As well as delaying the completion of the first brood, additional eggs prolong the period of high vulnerability to nest predation and brood parasitism [21]. In order to maximise overall productivity, birds could prioritise early completion of the first brood by maintaining typical clutch sizes. Increases in food availability earlier in the breeding season may result in higher breeding success and population growth by enabling earlier breeding and faster nestling growth, particularly if birds do not increase their clutch size. In the present study, we use a food supplementation experiment to measure the extent to which changes in food availability (as may occur in future climate scenarios) may drive changes in the different aspects of breeding behaviour that can affect overall demography.

Eurasian reed warblers, *Acrocephalus scirpaceus* (hereafter ‘reed warblers’) have an increasing West-European population and a northwest-advancing geographic distribution [30, 31]. This trend, linked with increasing spring temperatures suggests that, despite inhabiting a habitat with plentiful resources throughout the breeding season, reed warblers are limited during the very early stages, either by phenology of their prey, growth of nesting habitat or by weather conditions.

In this study, we test this limitation by experimentally increasing food availability (i.e. through the supply of more food) for a sample of breeding reed warblers, during the early stages of three breeding seasons, and compared the behaviour and breeding performance of this sample with that of a control group with natural food availability. Individual breeding pairs were supplemented with an unlimited food supply; they could not be provisioned with the additional amount of food predicted to occur under a particular climate projection. This was because multiple birds (and species) used the same supplementary feeders and so precisely regulating the amount of food for each pair was not feasible. The study should not, therefore, be viewed as an attempt to simulate future food availability. Rather, by *ad libitum* food supplementation during the early stages of the breeding season, at a time when the natural levels of food availability are gradually increasing, the food peak can be artificially and immediately advanced. This approach allows focussed investigation of the aspects of reed warbler behaviour and breeding performance that are currently limited by food availability during the early stages of the breeding season.

We assess how different aspects of breeding behaviour including laying date, clutch size, incubation period, nestling growth and first brood productivity responded to increasing food availability. We predict that: i) reed warblers will respond to increases in food availability by advancing their laying date and increasing nestling growth rates, in order to prioritise advancement of first brood completion; ii) clutch size will be unchanged in favour of maintaining a short period of incubation; and iii) the net effect of increased food availability will result in an increase in first brood productivity as well as earlier fledging of the first brood. Late completion of first broods may preclude the successful completion of second broods [32]. These mechanisms, linking protracted climate-driven increases in food supply across the spring in wetland habitats, to reed warbler breeding behaviour and productivity, may explain the current growth of reed warbler populations.

## Materials and Methods

### Ethics statement

The guidelines promoted by the Association for the Study of Animal Behaviour for the ethical use of animals in research are followed. The species caught as part of this study are common and not registered as an endangered or protected species in any country. All fieldwork was conducted after ethical approval from the British Trust for Ornithology ringing unit (Vafidis licence no; 5475; Facey licence no: 5411; Thomas licence no: 4127). Special approval from the animal ethics committee was not required for these activities as they are included on the authors’ bird ringing licences.

### Study sites

Two wetland locations in South Wales, UK, were used for the study. Cardiff Bay Wetland Reserve (CBWR; 51° 27’ 32” N, 3° 10’ 11” W) is a four-hectare wetland consisting of mixed scrub habitat, open pools and large areas of *Phragmites australis* reedbed. Cosmeston Lakes Country Park (CLCP, 51° 24′ 53″ N 3° 6′ 0″ W) supports two adjacent small reedbed sites (with a total area of approximately 1.5 hectares) separated by 200 m of freshwater lake habitat. Both sites are publicly owned and access was arranged through the local authority.

### Bird ringing / banding

Breeding adult reed warblers were monitored as part of an established monitoring project at the sites. All monitored pairs were caught during regular mist-netting sessions and fitted with a unique combination of three plastic colour rings and a numbered metal ring, to enable individual identification in the field.

### Food supplementation

Prey availability for reed warblers was experimentally increased across the two study sites over three breeding seasons by providing live and dried mealworms, *Tenebrio molitor* larvae (Coleoptera), in containers (2 litre-capacity) resting on small tables at 1.5 m height, from 1st of April onwards. Each study site supports three feeding stations, which were supplied with at least 200 mealworms (mean weight ± SD of mealworms = 112.00 ± 2.60 g, n = 100) and refilled every day (when required), until the end of the breeding season (September). Feeding bowls were enclosed in a wire mesh cage (measuring 300 x 200 x 200 mm of 10mm mesh) to prevent the mealworms being taken by carrion crows (*Corvus corvus*) and black-billed magpies (*Pica pica*), but permitting access to reed warblers. Each feeding station was regularly visited by between three and five pairs of colour-ringed breeding reed warblers each season (CBWR 2013–2014: 22 supplemented pairs out of 56 monitored territories; CLCP 2012–2013: 19 supplemented pairs out of 46 monitored territories). Other small passerines including European robins (*Erithacus rubecula*), common reed buntings (*Emberiza schoeniclus*) and Eurasian blue tits (*Cyanistes caeruleus*) occasionally gained access to the mealworms, but mealworms were always available for reed warblers using the feeders. All adults nesting within 50 m of the feeding stations discovered the mealworms within two to five days, and fed daily on the supplemented food from the laying period until the end of the breeding season in late July. Fed and control sites were located at least 150 m apart, to minimise the likelihood of accidental supplementary feeding of control birds visiting fed areas (no such incidences were observed). This was confirmed by individually identifying the adult reed warblers using the feeding stations and by remotely monitoring the stations with small video cameras (Sony Handycam DCR-SR32, Sony Corporation, http://sony.co.uk) and infrared-triggered trail cameras (Bushnell HD. http://Bushnell.co.uk). Activity around the feeding stations was recorded between 06:00 and 18:00 BST every two days throughout the breeding cycle. The positions of feeding stations were moved between successive breeding seasons to allow the treatment state of site-faithful individuals to change between years.

### Nest monitoring

Active nests (n = 102) were located by systematic searching of suitable nesting habitat (dense, tall stands of *Phragmites*) and by visually tracking adults back to the nest. The status of each nest was checked every two days until the first eggs were laid (in April and May), then they were checked twice a day (at ~10:00 and ~16:00 BST) until fledging (in June and July). In order to minimise disturbance to breeding birds and damage to *Phragmites* habitat, only first broods were monitored in this study.

The number of days since 1 April of that year was used as a measure of laying date earliness. The incubation period was defined as the time between the laying day of the last egg and the day of hatching of the first egg. Nestlings (at day four and day six after hatching) were weighed to 0.1 g using an electronic balance (Satrue SA-500). Since the nestlings were ringed on day six, it is not possible to assign growth rates to individual birds between day four and day six. Instead nestling weights were modelled using age and nest identity as independent variables to account for non-independence of measurements from the same age groups and nests. The effect of food supplementation on total first brood productivity was measured by comparing the mean number of chicks produced for each nest (including depredated nests and failures) in control and fed treatments. All nests in both treatment groups were filmed on at least three occasions during the nestling period, for up to two hours on each occasion, to confirm whether or not nestlings were being provisioned with mealworms as well as with other invertebrate taxa.

### Invertebrate monitoring

A measure of weekly invertebrate prey availability was determined at each site using double-sided yellow (dry-stick) invertebrate traps (measuring 50 mm^2^; Oecos Ltd, UK) set in eight locations across the reedbed habitat at a height of 50–120 cm (following Vafidis *et al*. 2014 [33]). Total weekly captures of Diptera, Aranea, Hymenoptera and Hemiptera, as well as other less-frequently encountered taxa (<1%) such as Coleoptera and Lepidoptera are recorded for each trap. The number of invertebrates captured by these traps reflects both their abundance and activity [34,35], so the mean weekly captures are used as an invertebrate “activity-density” to reflect the availability of naturally occurring prey for foraging reed warblers.

### Phragmites height

The mean total height of all *Phragmites* stems (from ground level to the top of the uppermost panicle) supporting each nest when the first egg was laid was measured using a fixed measuring rod. In order to obtain a measure of the status of *Phragmites* growth at the time of egg-laying, the mean total height is transformed into positive or negative differences of the overall mean total height of all measured stems (i.e. ‘*Phragmites* height index’).

### Weather variables

Mean air temperature, mean wind speed and total rainfall data for each day were summarised from measurements collected by an automated weather station (Davis Instruments Vantage Pro 2, Hayward, CA) located at 51° 27’ 17” N, 3° 9’ 45” W, 0.75 km and 4.5 km from CBWR and CLCP, respectively. Temperature measurements were summarised for the analysis to include mean temperature for all April measurements, the mean temperature during the egg laying period (the mean of air temperature measurements taken in the five days leading up to the production of the first egg for each nest), mean temperature during the incubation period (the mean of air temperature measurements during the incubation period for each nest) and mean temperature during the nestling period (the mean of air temperature measurements during the six days after hatching for each nest). Total rainfall (in mm) was the total measure of all the rainfall measurements taken during the laying, incubation or nestling periods (as specified above for mean temperature), for each nest. Likewise, wind speed (m/sec^-1^) was the mean wind speed value for the laying, incubation or nestling periods for each nest.

### Statistical analysis

This study tests the effects of food treatment on reed warbler laying date, clutch size, incubation duration, hatching success, rates of nestling growth, and overall first brood productivity (of all nesting attempts). As well as the availability of food, these reproductive parameters may also be affected by factors such as local weather conditions, and by other unmeasured constraints which may vary by year and site (e.g. competitive pressure, predator density).

These effects are investigated using the R statistical software, version 3.2.2 [36] fitting generalised linear models (GLMs) and generalised linear mixed-effects models (GLMMs) using the R package ‘lme4’ [37] (Table 1). Where appropriate, the identity number for each nest is used as a random effect in mixed-effects models to account for repeated measures (e.g. nestling measurements) from the same nest. The final models are selected using backward deletion of non-significant (P> 0.05) terms. The rates of predation are compared between fed and unfed treatments using a Fisher’s exact test due to the low instances of predation in the treatment group. Data exploration and model validation procedures follow the protocol described in Zuur *et al*. 2007 and Thomas *et al*. 2015 [38–39]. Outliers are identified using Cleveland plots, and collinearity is assessed using pair plots and variance inflation factor values. Homogeneity of variance is assessed by plotting the model residuals against the fitted values, and normality of residuals is assessed using histogram and QQ plots. Finally, influential observations are assessed using Cooks’ distance values.

**Table 1:** Starting models of GLM and GLMMs relating breeding performance parameters of reed warblers monitored in Cardiff Bay Wetland Reserve and Cosmeston Lakes Country Park in South Wales, UK, between 2012 and 2014.

Model	Sampling unit	Error family/link	Variables
**Laying date**	Nest	Guassian/identity	✓ Treatment (fed/control ✓Site ✓Invertebrate availability ✓Mean temperature ✓Mean April temperature ✓Mean wind speed ✓Total rainfall ✓Phragmites height ✓All two-way interactions
**Clutch size**	Nest	Poisson/log	✓ Treatment ✓Site ✓Laying date ✓Invertebrate availability ✓Mean temperature ✓Mean April temperature ✓Mean wind speed ✓Total rainfall ✓All two-way interactions
**Incubation duration**	Nest	Guassian/identity	✓ Treatment ✓Site ✓Clutch size ✓Incubation date ✓Invertebrate availability ✓Mean temperature ✓Mean April temperature ✓Mean wind speed ✓Total rainfall ✓All two-way interactions
**Hatching success**	Nest	Binomial/ logit	✓ Treatment ✓Clutch size ✓Laying date ✓Site ✓Invertebrate availability ✓Mean temperature ✓Mean April temperature ✓Mean wind speed ✓Total rainfall ✓All two-way interactions
**Nestling growth**	Individual nestling	Guassian/identity	✓ Treatment ✓Nestling age ✓Time of day ✓Brood size ✓Site ✓Incubation duration ✓Hatch date ✓Invertebrate availability ✓Mean temperature ✓Mean wind speed ✓Total rainfall ✓All two-way interactions
**Total 1^st^ brood productivity**	Nest	Poisson/log	✓ Treatment ✓Site ✓Invertebrate availability ✓Mean temperature ✓Mean wind speed ✓Total rainfall

## Results

Between 2012 and 2014, 200 breeding adult reed warblers, 102 nests, 416 eggs and 324 nestlings were monitored in CBWR and CLCP. With these data it is possible to examine how increases in food availability during the early stage of the breeding season affect different reproductive parameters of breeding reed warblers.

### Laying date

**Fig 1:**
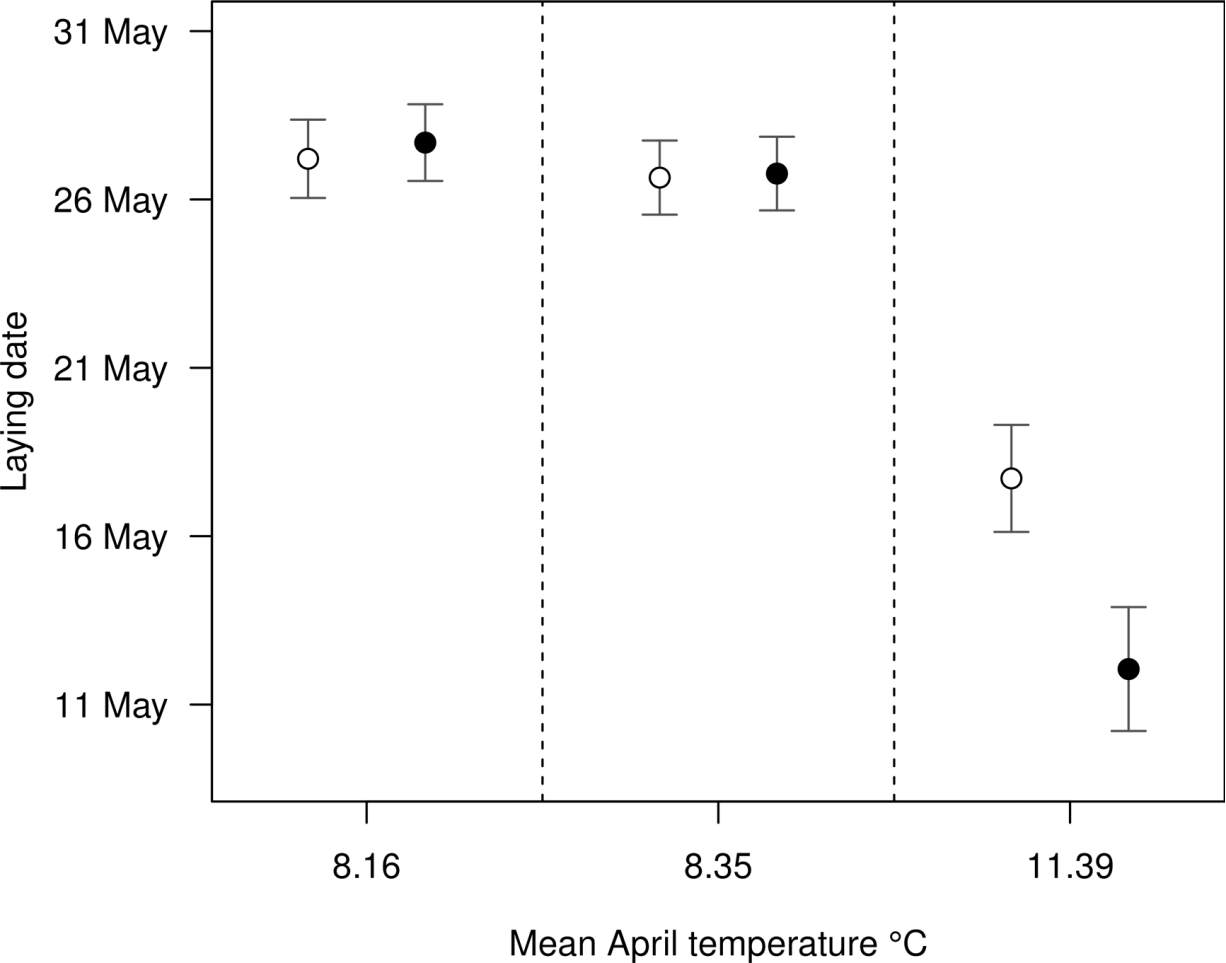
The effect of treatment and mean April temperature on reed warbler laying date (± SE). Control (unfed) nests are represented by unfilled circles and fed nests are represented by filled circles.

Mean April temperatures, rain, invertebrate availability and reed growth are significant predictors of the date of laying (adj R^2^ = 0.63, *F*_6,70_ = 22.88, *P*<0.0001). Earlier laying dates are associated with warmer mean April temperatures (-2.94 ± 0.60; *F*_1,75_ = 75.03; *P*<0.0001) and higher rainfall (-0.04 ± 0.01; *F*_1,74_ = 40.56; *P*<0.0001), while later breeding dates are associated with greater invertebrate availability (0.27 ± 0.11; *F*_*1*,*73*_ = 5.85; *P* = 0.018; Table 2). The effect of fed treatment was dependant on April temperatures, with each degree increase representing an advance by 1.90 days (± 0.88; *F*_*1*,*70*_ = 4.71, *P =* 0.0333; Fig 1). The mean difference in laying date between fed and control groups on the warmest recorded mean April temperature of 11.39°C is 5.66 days (± 0.30).

**Table 2:** GLM predicted effects of supplementary feeding treatment and environmental variables on laying date in reed warblers.

Parameter	Estimate ± SE	*F*	*P*
April temp	-2.94 ± 0.60	75.03	<0.0001
Rain	-0.04 ± 0.01	40.56	<0.0001
Invert avail.	0.27 ± 0.11	5.85	0.0181
Reed height	0.11 ± 0.05	10.35	0.0020
Treatment	16.00 ± 7.97	0.78	0.3796
Treatment × April temp	-1.90 ± 0.88	4.71	0.0333

### Clutch size, incubation duration and hatching success

Of the 102 nests monitored, only 80 completed their clutch, with the other 20 completely depredated, abandoned or destroyed by bad weather before clutch completion. Most completed clutches (69) contained a total of four eggs, ten clutches contained five eggs and one clutch contained three eggs. Clutch size is uncorrelated with treatment (*z* = -0.03; *P* = 0.1990), laying date (*z* = -0.05; *P* = 0.9780) or any environmental variables (P>0.8). The incubation period varies between nine and 13 days and is strongly influenced by clutch size, invertebrate availability, and total rainfall (Adj R^2^ = 0.34, *F*_6,70_ = 7.46; *P*<0.0001; Table 3). Each additional egg in the clutch increases the incubation duration by 1.01 days (± 0.07; *F*_1,71_ = 18.06; *P*<0.0001). Increases in incubation duration are correlated with total rainfall (0.04 days ± 0.01; *F*_1,72_ = 10.91; *P* = 0.0015) and invertebrate availability (0.01 days ± 0.00; *F*_1,74_ = 5.41; *P* = 0.0229). There is a significant interaction between treatment and mean temperature, with increasing temperatures corresponding with shorter incubation durations in the treatment group (-0.17 days ± 0.07; *F*_1,70_ = 6.47; *P* = 0.0132). Of the 102 nests monitored across the three breeding seasons, 80 nests resulted in successful hatching of at least one egg. Of these successful nests, 14 contained unhatched eggs; overall 313 eggs hatched out of 329. Hatching success is uncorrelated with treatment (LRT = 0.07; *P* = 0.7891), laying date (LRT = 1.03; *P* = 0.3089) and any environmental variables (*P*> 0.4).

**Table 3:** GLM Predicted effects of supplementary feeding treatment and environmental variables on incubation duration in reed warblers.

Parameter	Estimate ± SE	*F*	*P*
Clutch size	1.01 ± 0.25	18.06	<0.0001
Rain	0.04 ± 0.01	10.91	0.0015
Invert avail.	0.01 ± 0.00	5.41	0.0229
Mean temperature	0.03 ± 0.05	1.12	0.2944
Treatment	1.71 ± 0.82	2.78	0.0997
Treatment × mean temp	-0.17 ± 0.07	6.47	0.0132

### Nestling mass and predation

Treatment, brood size, nestling age, site and invertebrate availability are all significant predictors of nestling mass (Table 4). The mean difference in nestling mass between day four and day six is 2.90 g (± 0.10 g; *F*_1,455_ = 1520.74; *P*<0.0001). Supplemented nests are associated with heavier six-day-old nestlings (0.50 ± 0.16 g; *F*_1,457_ = 6.79; *P* = 0.0094). Brood size is also a highly significant predictor of nestling mass, with larger broods containing lighter nestlings (-0.52 ± 0.09; *F*_1,456_ = 35.93, *P*<0.0001). There is a significant and substantial effect of treatment on nest predation, with control nests suffering 21 predation events out of 61 nests (34%), and fed nests suffering one predation event out of 41 nests (2.5%; Fisher’s exact test; odds ratio = 0.07, *P<*0.0001). The 21 depredated control nests suffered complete predation of eggs, while the single depredated fed nest suffered the loss of two eggs (retaining three successfully hatched eggs). The study did not record any incidences of nestling predation from either treatment group.

**Table 4:** GLMM predicted effects of supplementary feeding treatment, age, brood size and environmental variables on nestling mass in reed warblers.

Parameter	Estimate ± SE	*F*	*P*
Treatment (Fed)	0.14 ± 0.13	41.21	<0.0001
Brood size	-0.52 ± 0.09	35.93	<0.0001
Nestling Age	2.90 ± 0.10	1520.74	<0.0001
Site	-0.30 ± 0.08	42.46	<0.0001
Invertebrate availability	-0.004 ± 0.001	9.62	0.0028
Treatment × Nestling age	0.50 ± 0.16	6.79	0.0112

### Total first brood productivity

The mean first brood productivity is 3.18 (± 0.15) chicks per pair. Treatment, site, total rain, and mean April temperature are all significant predictors of first brood productivity (adj R^2^ = 0.42; *F*_4,95_ = 19.28; *P*<0.0001; Table 5), with fed nests producing 1.14 (± 0.26; *F*_1,98_ = 29.18; *P* <0.0001) more chicks than control nests. Productivity was higher in warmer mean April temperature (0.42 chicks ± 0.11; *F*_1,97_ = 12.21; *P*<0.0001), while lower productivity was associated with higher rainfall (-0.04 chicks ± 0.01; *F*_1,95_ = 24.99; *P*<0.0001).

**Table 5:** GLM predicted effects of supplementary feeding treatment, site and environmental variables on total first brood productivity in reed warblers.

Parameter	Estimate ± SE	*F*	*P*
Treatment	1.14 ± 0.26	29.18	<0.0001
April temp	0.42 ± 0.11	12.21	0.3716
Site	-1.53 ± 0.30	10.74	<0.0001
Rain	-0.04 ± 0.01	24.99	<0.0001

## Discussion

Using supplementary feeding, this study investigates how changes in food availability affect different reproductive parameters that, together, influence the breeding success of reed warblers. Fed birds have earlier laying dates, higher nestling masses, and more fledged young. Incubation period was shorter in fed treatment groups when the mean April temperatures were warmest. In contrast, food supplementation does not measurably influence clutch size or hatching success.

### Laying date

Supplemental food advances the laying date by up to 5.6 (± 0.3) days during the warmest mean April temperatures. These results are consistent with observational studies of reed warblers [31, 40], where earlier breeding was associated with warmer May to July temperatures. Continental European populations of reed warbler have been reported to be laying 18 days earlier over the last 33 years [31, 41], but it has been unclear whether the primary driver behind earlier laying is the faster growth of nesting vegetation, or the earlier rise in availability of invertebrate prey. The relationship identified between later laying dates and higher values of invertebrate availability and reed height is likely to be correlated with time rather than more invertebrate prey and higher reeds driving later breeding. The significant food supplementation effect on laying phenology in our study suggests that laying date is constrained by food availability, as birds in both treatments were exposed to the same *Phragmites* growth conditions. It is likely that reed warblers may only be able to advance their laying date in response to increased food availability if reed growth phenology (which is limited by spring temperatures [42]) has advanced sufficiently to provide habitat suitable for earlier nest building.

Consistent with our findings, experimental increases in food availability are associated with earlier breeding dates in other passerine birds, including Eurasian blue tit [43]; great tit, *Parus major* [44], common starling, *Sturnus vulgaris* [45]; and European blackbird, *Turdus merula* [46]. Many of these species rely on highly seasonal prey resources (e.g. caterpillars) and therefore risk mismatch between supply and demand if they nest too late. This is less likely for reed warblers, which feed their young on a diverse range of invertebrate prey populations which emerge sequentially throughout the summer, resulting in a prolonged period of high abundance without any clear peak [47–50]. Instead, reed warblers may be taking advantage of the higher abundance of invertebrates and earlier reed growth under warmer spring conditions to maximise fitness of their offspring by breeding earlier, enabling longer periods to acquire experience in foraging before autumn migration [21,31,51,52] or creating the opportunity to initiate a second brood earlier in the breeding season.

### Clutch size

Clutch size in reed warblers appears to be less variable than in many other passerine species [53]. This may represent the outcome of a trade-off between current reproductive effort and future reproduction and survival [54]. Maintaining small clutch sizes reduces the period of egg-laying, as well as the duration required for incubation and nestling periods [55]. The advantages of this include reducing the period of vulnerability to predators and brood parasites [56], as well as leading to the earlier initiation of subsequent broods. Such benefits may outweigh the gains of increasing the clutch size of the first brood.

### Nest predation

Egg predation occurs much less frequently in supplementally fed nests than in control nests. Furthermore, the depredated control nests were subject to complete predation of the whole clutch, while the one supplemented nest only lost two eggs to predation. This difference may be explained by different nest attendance rates between treatment groups, with supplemented parent reed warblers spending less time foraging, reducing the frequency and duration of eggs being undefended, and uncovered by the well-camouflaged parent [57,58]. Lower rates of nest predation have been found in species that incubate continuously, such as mourning doves, *Zenaida macroura* [59], common eiders, *Somateria mollissima* [60], and common pheasants, *Phasianus colchicus* [61], as a strategy to reduce the period of nesting to minimise vulnerability to predation and maximise opportunities to multi-brood. As no nestling predation was observed in this study, the egg-laying and incubation stage appears to be the period most vulnerable to nest predation. Having sufficient food supplies during this period may well represent a means to minimise substantial losses to predation.

### Nestling growth

Supplementary feeding of parent reed warblers resulted in an increased rate of nestling growth. Nestlings of food-supplemented parents weighed 0.5 g (± 0.2) more at day six than nestlings of unsupplemented parents, which could be explained by more rapid growth or heavier stomach contents due to earlier feeding before being measured [62]. Increases in food abundance, parental investment and predation have all been shown to modify nestling growth rate in *Acrocephalus* warblers [63–65]. Likewise, the offspring of supplementally fed great tits grew faster than control nestlings [66]. Greater prey availability for parents is therefore likely to increase nestling growth rates, which could accelerate fledging and thus reduce the period of vulnerability to predation and adverse weather conditions. Nestling reed warblers normally fledge after 10–11 days but are able to jump from the nest to evade predators from day seven [67]. Given the high rates of predation in wetlands [68], adaptations to reduce the period of nestling vulnerability are likely to be strongly favoured by selection.

## Conclusions

European wetlands currently provide abundant prey resources for breeding wetland migrants, but it is predicted that climate warming will further increase overall prey abundance [42], and that higher abundances of prey will become available earlier in the breeding season. To investigate the effects of such climate-driven increases and phenological advances in food availability, in the present study individual breeding pairs received either an unlimited food supply or natural food availability under current climate conditions. The results suggest that reed warblers advance the initiation of first broods in response to earlier onset of suitable foraging and nesting conditions, whereas clutch size, incubation duration and hatching success were not directly affected by additional food. First brood productivity was higher in food supplemented treatment groups, which is due to lower rates of predation. Further research is needed to test the possibility that offspring survival and recruitment may be increased through lower predation and earlier fledging of supplemented nests. Despite their access to extensive periods of abundant invertebrate food throughout the breeding period, the success rate of subsequent breeding attempts in reed warblers is low (≤30% [30, 31, 41, 52, 69]. Our ongoing research efforts will focus on whether earlier completion of the first brood actually leads to greater chance of success in subsequent breeding attempts.
